# Manifestations of Perihepatic Lymph Nodes in Acute Flare of Chronic Hepatitis B: Association with HBeAg Status and with HBeAg Seroconversion

**DOI:** 10.1371/journal.pone.0117590

**Published:** 2015-02-17

**Authors:** Yen-Ling Ko, Chi-Shu Sun, Kun-Ming Chung, Yu-Min Lin, I-Che Feng, Ming-Jen Sheu, Lok-Beng Koay, Ching-Yih Lin, Chung-Han Ho, Hsing-Tao Kuo

**Affiliations:** 1 Department of Internal Medicine, Division of Hepatogastroenterology, Chi-Mei Medical Center, Tainan, Taiwan; 2 Department of Senior Citizen Service Management, Chia Nan University of Pharmacy & Science, Tainan, Taiwan; 3 Department of Internal Medicine, Division of General Internal Medicine, Chi-Mei Medical Center, Tainan, Taiwan; 4 Department of Medical Research, Chi Mei Medical Center, Yongkang, Tainan, Taiwan; 5 Department of Hospital and Health Care Administration, Chia Nan University of Pharmacy and Science, Tainan, Taiwan; Kaohsiung Medical University Hospital, Kaohsiung Medical University, TAIWAN

## Abstract

It has been observed that enlargement of perihepatic lymph nodes may be seen in patients with chronic hepatitis B, particularly during acute flares of CHB. We hypothesized that there may be a correlation between the nodal change patterns in CHB patients with acute flare and HBeAg status. Perihepatic lymph node sizes of 87 patients with acute flares of CHB were documented, with a median follow up of 43 months. Patients were separated into 3 groups, HBeAg-positive with HBe seroconversion (group 1), HBeAg-positive without HBe seroconversion (group 2), and HBeAg-negative (group 3). Group 1 has the highest incidence of enlarged lymph nodes (92.3%) compared with group 2 (75.8%) and group 3 (46.8%) (p = 0.003). And if nodal width at acute flare was > 8mm and interval change of nodal width was >3mm, the incidence of HBeAg seroconversion will be 75% (p<0.001).

**Conclusion:**

Larger perihepatic lymph nodes are seen in CHB acute flare patients with positive HBeAg and the magnitude of nodal width change may predict HBeAg seroconversion at recovery.

## Introduction

In the natural history of hepatitis B virus (HBV) infection via perinatal transmission, the point of hepatitis B envelope antigen (HBeAg) seroconversion is an important milestone. Long term follow up show chronic hepatitis B (CHB) infected individuals with HBeAg seroconversion have longer survival and less incidences of cirrhosis and hepatoma occurrence[1–4]. The estimated annual HBeAg seroconversion rate is 2–15%[5]. Most spontaneous HBeAg seroconversion occurs before age 40[6], those older than 40 years old have HBeAg seroconversion rates lower than 10% and higher rates of progression to cirrhosis[7]. Thus, the current guidelines in treatment of CHB patients further separate patients into different groups according to the HBeAg status and the achievement of HBeAg seroconversion is one of the major goals in HBeAg positive patients[8].

In the 1980s, Realdi[9] and Hoofnagle[10] have found that spontaneous HBeAg seroconversion in CHB patients is accompanied by a decrease in HBV DNA titers and alanine aminotransferase (ALT) levels, sometimes even disappearance of hepatitis B surface antigen (HBsAg). Furthermore, study by Liaw et al found that the occurrence of acute exacerbation, defined as an elevation of ALT >300IU/L, is a key factor in precipitating HBeAg seroconversion and CHB with acute exacerbation has similar biochemical and histological presentations as acute hepatitis B but with milder clinical manifestations that last longer in duration[11]. Review summary of previously known facts include those younger than 40 years old, horizontal transmissions, individuals infected at a later age, specific HLA genotypes, immunocompetent individuals, those infected with genotypes B or D, high ALT during CHB with acute exacerbation, α-fetoprotein (AFP) level >100ng/ml, serum DNA level <10^7^copies/mL, and presence of severe inflammation such as bridge hepatic necrosis during acute exacerbations are all factors favorable to HBeAg seroconversion[12–18].

Earlier studies demonstrated a high prevalence of perihepatic lymphadenopathy in patients with chronic hepatitis C (CHC) and other acute and chronic liver disease of viral-related or immune-related etiology. The accuracy of ultrasound detection of enlarged lymph nodes have been well correlated with concurrent examinations by CT and MRI. [19,20] The mechanism of perihepatic lymphadenopathy in CHC is unknown but appears to be related to viral replication within the liver and the host’s immune-mediated inflammatory response. Associations between perihepatic lymphadenopathy to serum parameters of cytolysis, severity of histologic damage, viremia, and a high CD8 lymphocyte level have been reported in some CHC studies[21–23]. There were few reports concerning perihepatic lymphadenopathy in patients with CHB. We had mentioned a correlation between the nodal size and ALT level in subjects of HBV infection[24]. The nodal change during CHB with acute exacerbation is interesting, and the relationship of the nodal size with HBeAg seroconversion has never been observed. We herein perform this prospective observational study in our clinical practice.

## Materials and Methods

### Patients and study design

We enrolled 102 patients diagnosed with CHB with acute exacerbation between June of 2003 to January of 2013 and simultaneously measuring the size of the perihepatic lymph nodes in this prospective cohort study. Patients with acute exacerbation of CHB were defined as positive HBsAg for at least 6 months with elevated ALT levels >5 times upper normal limit(>250IU/L) [25]. Those with superinfection with other virus and known alcohol consumption, malignancy, autoimmune disease and bacterial infection were excluded. A total of fifteen patients were excluded from the cohort study; twelve patients were excluded due to inadequate visualization of the lymph node and hepatic artery due to obesity or intestinal gas interference, three patients have missing data. The remaining 87 patients were separated into three groups according to their HBeAg status, group 1(N = 26): HBeAg-positive with HBeAg seroconversion during follow up, group 2(N = 29): HBeAg-positive without HBeAg seroconversion during follow up and group 3(N = 32): HBeAg-negative during acute exacerbation. The status of HBeAg seroconversion is defined as loss of HBeAg and presence of anti HBe antibody during follow up. Patients were followed up at clinic visits for 2–116 months (median 43 months) in an interval between one to six months based on clinical requirements. Twenty four (27.6%) patients had history of antiviral withdrawal about 1 to 8 months before the episode of acute exacerbation were termed withdrawal hepatitis flare. The other 63 (72.4%) patients who had no history of antiviral or immunosuppressive therapies were presumed as spontaneous hepatitis flares. Sixty-two (71.3%) patients were treated with antiviral therapies, either oral nucleos(t)ides analogues or pegylated- interferon, during the follow-up periods and the other 25 (28.7%) patients remained untreated. There was no mortality in this study cohort. Only 4 patients (one in group 1, one in group 2 and 2 in group 3) achieved HBsAg loss during follow-up periods. Ethics statement: This study was approved by Chi Mei Medical Center Institutional Review Board and ethics committee (approval number: IRB09712-013). All data were analyzed anonymously. No written or verbal consents were obtained from patients since this study was conducted via retrospective chart review method after prospectively enrolling eligible patients. All the biochemistry data and sonographic images were followed routinely as would be done in checkups of chronic hepatitis B patients and treatments were in accordance to current practice guidelines.

### Biochemistry and laboratory methods

The white blood cell (WBC) count, platelet (PLT) count, ALT level, total bilirubin level, AFP level were measured during the acute flare stage and during follow up as indicated, they were performed using routine automated techniques at our clinical pathology laboratories. Serum hepatitis markers including HBsAg, anti-HBs, HBeAg, anti-HBe, IgM anti HBc, anti-HDV, and anti-HCV were assayed using the EIA kit (Abbott Diagnostics, North Chicago, IL). HBV genotype was determined using PCR-restriction fragment length polymorphism of the surface gene of HBV. Serum HBV DNA was assayed using Roche Cobas Amplicor HBV Monitor test (Roche COBAS TaqManHBV Test, lower limit of detection: 69 or 1.84 log 10 copies/mL; 12 or 1.08 log 10 IU/mL, Roche Diagnostics, Pleasanton, CA).

### Ultrasound detection of perihepatic lymph node

After standard scanning of the whole abdomen was completed, any solid structures beside the common hepatic artery and/or the main portal vein were carefully studied. One or more ovoid masses less echogenic than the liver parenchyma are thought to be solid on Doppler sonography; these masses are clearly separate from adjacent organs and vessels on transverse, sagittal, and oblique scans. The maximum size of each lymph node was measured on the longest axis (length) and the corresponding perpendicular axis (width); the volume was calculated as a rotating ellipsoid with the formula of 〔4/3π × length/2 × (width/2)^2^〕. In subjects with multiple lymph nodes, the measured and calculated values were added for a total value, and the maximal values were used for analysis[24]. During the recovery stage, usually within 3–6 months after acute flare, when the ALT return to less than 5 times upper normal limit (ALT <2X in 87% subjects), the size of perihepatic lymph node was measured again. An enlarged lymph node was defined as the nodal width equal to or more than 5mm. The ultrasonographic liver parenchymal manifestation was categorized as early change (normal/ fatty liver) or late change (chronic liver parenchymal disease/liver cirrhosis).

### Statistical analyses

Comparisons between groups were made with Pearson's chi-square test or Fisher’s exact test for categorical variables and Kruskal-Wallis test or Wilcoxon rank sum test for continuous variables. For finding the independent factor, the univariate logistic regression analysis was used to select possible variables as p-value<0.1. Then, the multiple logistic regression analysis was used to identify the final model of the independent factors. A *p*-value <0.05 was considered significant. All analyses were presented using Statistical Analysis System (SAS) statistical software (version 9.2; SAS Institute, Inc, Cary, NC).

## Results

There were no differences between groups associated with the sex ratio, origins of acute exacerbation, treatment status during follow-up periods and the total follow-up time. The WBC and PLT count, total bilirubin level, AFP level at acute flare stage showed no differences between groups, however, ALT level and HBV DNA level were higher in group 1 and lower in group 3 (Table 1).

**Table 1:** The distributions of baseline characteristics and variable factors during acute flare including the results of nodal width and volume for 3 groups.

	Group1(N = 26)	Group2(N = 29)	Group3(N = 32)	p-value*
Sex, n (%)				
Male	10(38.46)	15(51.72)	15(46.88)	0.6104
Female	16(61.54)	14(48.28)	17(53.12)	
Age, median(IQR)	35.5(31–47)	40(32–45)	44.5(36.5–55.5)	0.0353
WBC(×10 ^3^/uL), median(IQR)	5750(4700–7000)	6100(4800–6800)	5900(5500–6500)	0.9066
Platelets (×10 ^3^ /uL), median(IQR)	206(140–258)	193(155–236)	185.5(157–247.5)	0.9401
ALT(IU/L), median(IQR)	1107(613–1802)	508(390–1071)	863(480–1692)	0.0369
BilT(mg/dL), median(IQR)	1.84(0.86–5.03)	0.93(0.68–1.77)	1.01(0.67–4.45)	0.1749
BilT>2(mg/dL), n (%)				
No	13(50.00)	23(79.31)	19(59.38)	0.0677
Yes	13(50.00)	6(20.69)	13(40.63)	
log VL, median(IQR)	7(7–8), 3 missing	7(5–8), 2 missing	6(5–7), 6 missing	0.0037
AFP(ng/mL), median(IQR)	13.95(3.89–18.83)	8.22(4.00–57.00)	8.12(5.64–13.30)	0.9879
WT(mm), median(IQR)	8.00(6.00–9.00)	6.10(4.70–7.00)	4.90(0.00–7.00)	0.0004
Vol(mm^3^), median(IQR)	37.70(33.28–243.36)	25.48(18.72–133.12)	18.11(0.00–93.96)	0.0113
dWT(mm), median(IQR)^＊＊^	3.00(1.30–5.00)	1.1(0.20–2.00)	0.20(0.00–1.00)	0.0002
dVol (mm^3^), median(IQR)^＊＊^	29.58(13.00–59.62)	10.36(0.00–50.44)	1.77(0.00–12.21)	0.0009
WT > = 5mm (%)	24/26(92.3)	22/29(75.8)	15/32(46.8)	0.0030
Liver state, n (%)				
normal/fatty	17(65.38)	22(75.86)	13(40.63)	0.0154
CLD/LC	9(34.62)	7(24.14)	19(59.37)	
Cause of acute flare, n (%)				
spontaneous hepatitis flare	20(76.92)	17(58.62)	26(81.25)	0.1178
withdrawal hepatitis flare	6(23.08)	12(41.38)	6(18.75)	
Treatment, n (%)				
nil	6(23.08)	7(24.14)	12(37.50)	0.3855
NUC/PEG	20(76.92)	22(75.86)	20(62.50)	
Follow up month, median(IQR)	55(20–65)	41(23–60)	30.5(14.0–72.5)	0.6369

Group 1: HBeAg-positive with HBeAg seroconversion, Group 2: HBeAg-positive without HBeAg seroconversion, Group 3: HBeAg-negative.

*p-value is from Kruskal-Wallis test for continuous variables and Pearson’s chi-square test for categorical variables.

^＊＊^ The nodal size change for both width (dWT) and volume (dVol) between acute flare stage and recovery stage.

Abbreviations: WBC = white blood cell count, ALT = alanine aminotransferase, AFP = alpha-fetoprotein, BilT = total bilirubin level, VL = viral load, CLD = chronic liver parenchymal disease, LC = liver cirrhosis both diagnosed as ultrasonographic criteria, NUC = nucleos(t)ide analogue, PEG = pegylated interferon, both current standard antiviral therapies for chronic B hepatitis, WT = nodal width, Vol = volume.

Most of the subjects presented with enlarged lymph nodes during acute flare stage (70% in total). Group 1 has the highest incidence of enlarged lymph nodes (92.3%) compared with groups 2 of 75.8%, and groups 3 with 46.8% (*p* = 0.003). The median nodal width (mm) and volume (mm^3^) in each groups are: group 1 (8.00/37.70), group 2(6.10/25.48) and group 3 (4.90/18.11) (*p* = 0.0004/*p* = 0.0113). The interval changes of nodal sizes (median, width/ volume) between acute flare stage and recovery stage are: group 1 (3.00/29.58), group 2 (1.1/10.36) and group 3 (0.20/1.77) (*p* = 0.0002/*p* = 0.0009). There are significant differences between the groups in the nodal size at acute flare stage and the interval nodal changes between acute flare stage and recovery stage (Table 1). The ultrasound manifestations of liver parenchyma are earlier and finer in group 1 and 2 than those of group 3. There are no differences between groups regarding the causes of acute exacerbation, treatment status and follow up time (Table 1).

Differences observed between HBeAg-positive (group 1 and 2) vs. HBeAg-negative (group 3) are: In univariate analysis, HBeAg-positive patients were younger (p<0.01) with more early liver parenchymal change (p<0.0055), higher HBV DNA level (p<0.005) and larger nodal size (p<0.001) as compared with HBeAg-negative patients (Table 2). There are no differences between sex ratio, WBC, PLT, ALT, total bilirubin, AFP and no difference between the cause of acute exacerbation. During follow-up, the proportion of subjects receiving antiviral therapy and follow up time were comparable between groups but the interval nodal changes between acute flare stage and recovery stage were more prominent in HBeAg-positive group (p<0.001) (Table 2). Multiple logistic regression showed HBV DNA level (p<0.0168) and nodal width at acute flare stage (p<0.0253) were the only two significant factors (Table 3) that differ in these two groups.

**Table 2:** Distribution for CHB w AE eAg+ vs. CHB w AE eAg- including the results of nodal widths.

	Group1/ Group2 (N = 55)	Group3 (N = 32)	p-value*	Odds ratio (95% C.I.)
Sex, n (%)				
Female	30(54.55)	17(53.12)	0.8980	1.00(ref.)
Male	25(45.45)	15(46.88)		1.06(0.44–2.54)
Age, median(IQR)	36(31–46)	44.5(36.5–55.5)	0.0099	0.95(0.91–0.99)
WBC(×10 ^3^/uL), median(IQR)	5900(4700–7000)	5900(5500–6500)	0.7280	1.00(0.98–1.02)
PLT(x10^3^/uL), median(IQR)	194(151–258)	185.5(157–247.5)	0.8637	1.00(0.99–1.01)
ALT(IU/L)/100, median(IQR)	7.01(4.35–14.08)	8.63(4.80–16.92)	0.4105	0.98(0.93–1.02)
BilT(mg/dL), median(IQR)	1.11(0.75–3.14)	1.01(0.67–4.45)	0.8776	0.97(0.90–1.04)
BilT>2(mg/dL), n (%)				
No	36(65.45)	19(59.38)	0.5707	1.00(ref.)
Yes	19(34.55)	13(40.63)		0.77(0.31–1.89)
log VL, median(IQR)	7(6–8), 5 missing	6(5–7), 6 missing	0.0051	1.61(1.17–2.23)
AFP(ng/mL), median(IQR)	10.30(3.89–40.80)	8.12(5.64–13.30)	0.8880	1.00(0.99–1.00)
WT(mm), median(IQR)	7.0(5.0–8.8)	4.9(0.0–7.0)	0.0011	1.27(1.09–1.48)
Liver state, n (%)				
normal/fatty	39(70.91)	13(40.63)	0.0055	1.00(ref.)
CLD/LC	16(29.09)	19(59.37)		0.28(0.11–0.70)
Treated condition, n (%)				
spontaneous hepatitis flare	37(67.27)	26(81.25)	0.1596	1.00(ref.)
withdrawal hepatitis flare	18(32.73)	6(18.75)		2.11(0.74–6.03)
Treatment, n (%)				
nil	13(23.64)	12(37.50)	0.1682	1.00(ref.)
NUC/PEG	42(76.36)	20(62.50)		1.94(0.75–5.00)
Follow month, median(IQR)	50(22–65)	30.5(14.0–72.5)	0.3599	1.00(0.99–1.02)

Group 1 and 2: HBeAg-positive and group 3: HBeAg-negative.

*p-value is from Wilcoxon rank sum test for continuous variables and Pearson’s chi-square test for categorical variables.

Abbreviations: WBC = white blood cell count, ALT = alanine aminotransferase, AFP = alpha-fetoprotein, BilT = total bilirubin level, VL = viral load, CLD = chronic liver parenchymal disease, LC = liver cirrhosis both diagnosed as ultrasonographic criteria, NUC = nucleos(t)ide analogue, PEG = pegylated interferon, both current standard antiviral therapies for chronic B hepatitis, WT = nodal width.

**Table 3:** The multiple logistic regression analysis for variables between HBeAg-positive and HBeAg-negative.

	Odds ratio	p-value
Age	1.01(0.95–1.07)	0.7958
Log VL	1.52(1.08–2.13)	0.0168
WT	1.25(1.03–1.51)	0.0253
Liver state		
normal/fatty	1.00(ref.)	
CLD/LC	0.29(0.08–1.00)	0.0505

Abbreviations: VL = viral load, WT = nodal width, CLD = chronic liver parenchymal disease, LC = liver cirrhosis.

**Fig 1:**
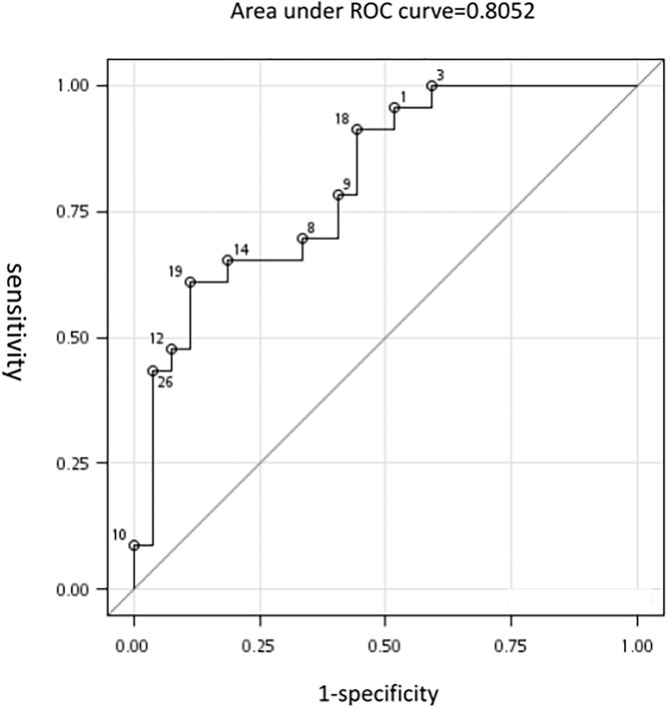
The ROC curve of nodal width for predicting HBeAg seroconversion in chronic B hepatitis with acute flare. The area under curve is 0.8052.

**Fig 2:**
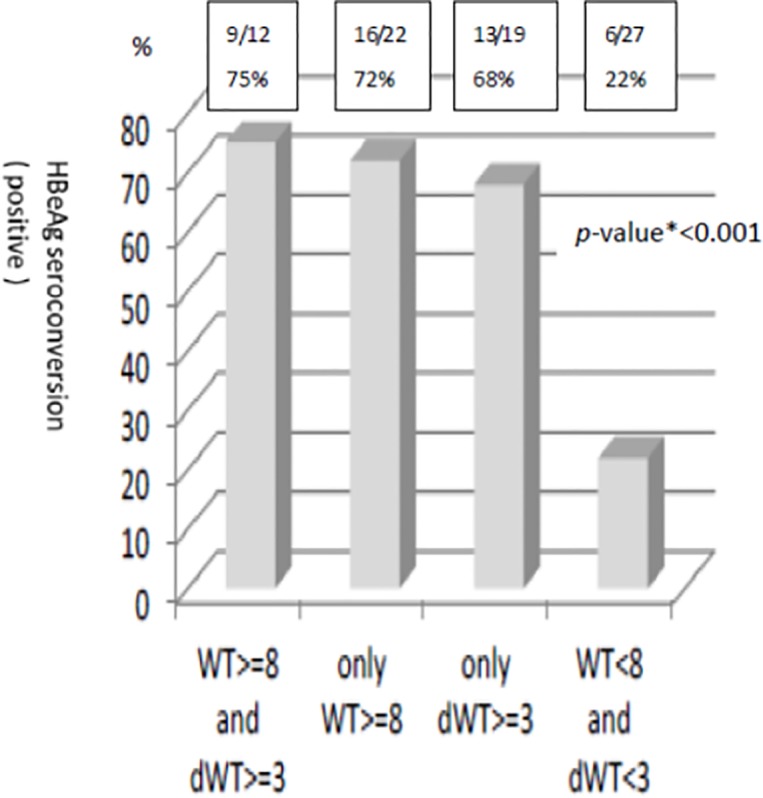
The power of combining nodal width (WT) at acute flare stage and nodal change (dWT) between acute flare and recovery stage to predict HBeAg seroconversion. *p-value is from Pearson’s chi-square test.

Differences between HBeAg-positive with HBeAg seroconversion (group 1) vs. HBeAg-positive without HBeAg seroconversion (group 2) include: For those of HBeAg-positive with acute exacerbation, ALT level, total bilirubin level, HBV DNA level and nodal size at acute flare stage were significantly higher in those with HBeAg seroconversion than those without (Table 4). After adjustment with multiple variables, the logistical regression analysis revealed higher HBV DNA level was a significant factor for e-seroconversion (p<0.01, OR 2.07, 1.16∼3.69) and the nodal size (width) at acute flare stage was a borderline significant factor for HBeAg seroconversion (p = 0.053, OR 1.26, 1.00∼1.58) (Table 5). The area under receiver operating characteristic curve of nodal width for predicting HBeAg seroconversion in chronic B hepatitis with acute flare is 0.8052 (Fig. 1). If the nodal width at acute flare stage was over 8mm and the interval change of nodal width over 3mm, the incidence of HBeAg seroconversion will be 75% (p<0.001) (Fig. 2).

**Table 4:** The distributions of baseline characteristics and variable factors during acute flare including the results of nodal width for group 1 and 2.

	Group1(N = 26)	Group2(N = 29)	p-value*	Odds ratio (95% C.I.)
Sex, n (%)				
Male	10(38.46)	15(51.72)	0.3240	1.71(0.59–5.02)
Female	16(61.54)	14(48.28)		
Age, median(IQR)	35.5(31–47)	40(32–45)	0.8993	1.00(0.95–1.05)
WBC(x10^3^/uL), median(IQR)	5750(4700–7000)	6100(4800–6800)	0.7808	1.00(0.98–1.03)
PLT(x10^3^/uL), median(IQR)	206(140–258)	193(155–236)	0.7873	1.00(0.99–1.01)
ALT(IU/L)/100, median(IQR)	1107(613–1802)	508(390–1071)	0.0135	1.05(0.98–1.13)
BilT(mg/dL)	1.84(0.86–5.03)	0.93(0.68–1.77)	0.0567	1.08(0.95–1.23)
BilT>2(mg/dL), n (%)				
No	13(50.00)	23(79.31)	0.0225	3.83(1.18–12.50)*
Yes	13(50.00)	6(20.69)		
log VL, median(IQR)	7(7–8), 3 missing	7(5–8), 2 missing	0.0650	1.57(1.01–2.46)*
AFP(ng/mL), median(IQR)	13.95(3.89–18.83)	8.22(4.00–57.00)	0.8265	0.99(0.98–1.01)
WT(mm), median(IQR)	8.00(6.00–9.00)	6.10(4.70–7.00)	0.0283	1.28(1.02–1.60)*
Liver state, n (%)				
normal/fatty	17(65.38)	22(75.86)	0.3930	1.66(0.52–5.38)
CLD/LC	9(34.62)	7(24.14)		
Treated condition, n (%)				
spontaneous hepatitis flare	20(76.92)	17(58.62)	0.1487	0.43(0.13–1.38)
withdrawal hepatitis flare	6(23.08)	12(41.38)		
Treatment, n (%)				
nil	6(23.08)	7(24.14)	0.9263	1.06(0.31–3.69)
NUC/PEG	20(76.92)	22(75.86)		
Follow month, median(IQR)	55(20–65)	41(23–60)	0.6733	1.00(0.98–1.02)

Group 1: HBeAg-positive with HBeAg seroconversion and group 2: HBeAg-positive without HBeAg seroconversion.

*p-value is from Wilcoxon rank sum test for continuous variables and Pearson’s chi-square test for categorical variables.

Abbreviations: WBC = white blood cell count, ALT = alanine aminotransferase, AFP = alpha-fetoprotein, BilT = total bilirubin level, VL = viral load, CLD = chronic liver parenchymal disease, LC = liver cirrhosis both diagnosed as ultrasonographic criteria, NUC = nucleos(t)ide analogue, PEG = pegylated interferon, both current standard antiviral therapies for chronic B hepatitis, WT = nodal width.

**Table 5:** The multiple logistic regression analysis for variables between group 1 and 2.

	Odds ratio	p-value
Log VL	2.07(1.16–3.69)	0.0133
WT	1.26(1.00–1.58)	0.0531
BilT	1.08(0.88–1.34)	0.4488
ALT/100	1.06(0.96–1.17)	0.2574
Age	1.03(0.96–1.10)	0.3834
Sex		
Female	1.00(ref.)	
Male	0.91(0.22–3.76)	0.8967
Treat condition		
Spontaneous hepatitis flare	1.00(ref.)	
Withdrawal hepatitis flare	0.63(0.15–2.64)	0.5270

Group 1: HBeAg-positive with HBeAg seroconversion and group 2: without HBeAg seroconversion.

Abbreviations: VL = viral load, WT = nodal width, BilT = total bilirubin level, ALT = alanine aminotransferase.

### Nodal enlargement in subgroups

We also studied the incidence of nodal enlargement (nodal width > = 5mm) according to the etiology of acute exacerbation; either spontaneous hepatitis flare or withdrawal hepatitis flare. In HBeAg positive patients who achieved HBeAg seroconversion, those of spontaneous hepatitis flare had higher incidence of enlarged node than those of withdrawal hepatitis flare (*p* <0.05) (Table 6).

**Table 6:** The incidence of nodal enlargement (width > = 5mm) in subgroup analysis.

Groups/Subgroups	Spontaneous hepatitis flare	Withdrawal hepatitis flare	*p* value*
Group 1			
> = 5mm	20(83%)	4(17%)	0.046*
<5mm	0(0%)	2(100%)	
Group 2			
> = 5mm	13(59%)	9(41%)	1.000
<5mm	4(57%)	3(43%)	
Group 3			
> = 5mm	12(80%)	3(20%)	1.000
<5mm	14(82%)	3(18%)	

*p-value is from Fisher's exact test.

### Validation cohort

We picked out another cohort of HBeAg positive patients (N = 29) with chronic hepatitis B acute exacerbation to validate the predictive ability of lymph node width (LN WT) change in size to indicate later HBeAg seroconversion. There were 14 patients with subsequent e-seroconversion and 15 patients without e-seroconversion (Table 7).

**Table 7:** The differences of clinical parameters and nodal manifestations between study and validation group.

	Study Group (N = 55)	Validation Group (N = 29)	p value
Age, medium(IQR)	36(31–46)	41(36–44)	0.40
e SC+ t to eSC medium (IQR)	11M (6–16) (N = 26)	8M (3–12) (N = 14)	0.31
e SC- t to FU medium (IQR)	41M (23–60) (N = 29)	24M (22–30) (N = 15)	0.02
eSC+ WT medium (IQR)	8.00(6.00–9.00)	5.20(4.50–5.90)^＊^	0.016
eSC+ dWT medium (IQR)	3.00(1.30–5.00)	4.10(1.60–5.50)^＃^	0.65
eSC- WT medium (IQR)	6.10(4.70–7.00)	6.00(2.80–7.00)^＊^	0.39
eSC- dWT medium (IQR)	1.1(0.20–2.00)	1.30(0.00–1.60)^＃^	0.41

＃: p = 0.0011

＊: p = 0.69.

Abbreviations: e SC+: positive HBeAg seroconversion, t to eSC: time from acute flare to HBeAg seroconversion, eSC-: negative HBeAg seroconversion, t to FU: time from acute flare stage to end of follow up, WT: nodal width, dWT: The nodal size change for nodal width between acute flare stage and recovery stage.

Validation group patients’ ages are comparable to those of the tested group patients without statistical significance. However, most of the validation cohort of patients were enrolled after 2011, thus the follow up time to e-seroconversion after acute exacerbation were shorter in average compared to the tested group of patients. The sizes of the LN WT were not significantly different between those with and without e-seroconversion, but the change in lymph node width between those in the acute exacerbation stage and those in the remission stage is significantly different. Our validation cohort shows that lymph node size(WT) ≧ 8mm or interval change (dWT) ≧ 3mm is still able to predict e-seroconverson within 12 months of acute exacerbation (Table 8).

**Table 8:** The power of nodal width>8mm at acute flare and nodal change>3mm between acute flare and recovery stage to predict HBeAg seroconversion in study and validation group.

Study Group	WT> = 8mm or dWT> = 3mm	WT> = 8mm and dWT> = 3mm	Only WT> = 8mm	Only dWT> = 3mm	WT<8mm and dWT<3mm	*p*-value*
e SC+ N = 26	20	9	16	13	6	<0.001
e SC N = 29	6	3	6	6	21	
Percentage	76.92	75	72.72	68.42	22.22	
Validation Group						
e SC+ N = 14	9	2	2	9	5	<0.001
e SC- N = 15	1	0	1	0	14	
Percentage	90	100	66.66	100	26.31	

Abbreviations: e SC+: positive HBeAg seroconversion, eSC-: negative HBeAg seroconversion, WT: nodal width, dWT: The nodal size change for nodal width between acute flare stage and recovery stage.

*p-value is from Pearson’s chi-square test.

**Fig 3:**
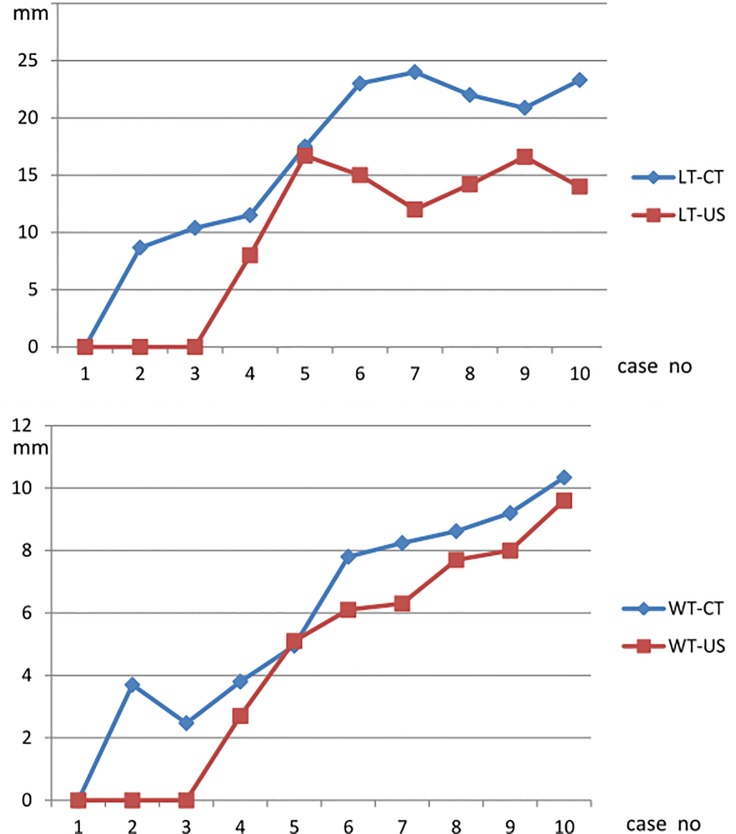
Validation for nodal length values measured by ultrasound (LT-US) with computed tomography (LT-CT). Validation for nodal width values measured by ultrasound (WT-US) with computed tomography (WT-CT).

We also looked at 10 patients with chronic hepatitis or cirrhosis who also underwent abdominal CT survey of liver due to sonographic suspicion of hepatic nodular lesion and concurrently checking the perihepatic lymph nodes sizes via sonographic measurements and comparing the CT measurements of perihepatic lymph nodes sizes and found that lengths and widths results were comparable in both image modalities (Fig. 3). However, the lymph node sizes measured via the CT images were generally larger than the measurements obtained via sonography, which may be due to both partial volume effect of the CT scan and an angle around 30–60 degrees present between the nodal plane and body’s coronal plane. Meanwhile, sonographic survey of lymph nodes with length <10mm and width <3mm may not be as reliable as CT exam due to the inherent lower resolution of sonographic images.

## Discussion

To our knowledge, this is the first published study on the manifestations of perihepatic lymph node in chronic hepatitis B with acute exacerbation. We found that most of the lymph nodes in CHB patient will enlarge in the acute exacerbation phase with significant difference in various clinical situations. Firstly, HBeAg positive group has larger nodal size than HBeAg negative group; secondly, HBeAg positive with seroconversion group has larger nodal size than those without seroconversion.

According to Sherlock et al in 1972, the inflammatory reaction of hepatitis B resulted from the host immune response[26] and HBeAg seroconversion often occurs in the immune clearance phase in the natural course of the virus [11,12]. Further study in CHB with acute exacerbation found that hepatitis B core antigen (HBcAg) might be a target of cytotoxic T lymphocytes, and expression of liver cell membrane HLA-1 also plays a role in promoting hepatocytolysis [27,28]. Tsai et al applied peripheral blood mononuclear cells (PBMC) to perform immunological test before and after acute exacerbation and found that increased T cell responses to HBcAg/HBeAg occurred before HBeAg seroconversion, but no changes were seen in HBsAg carriers[29]. Numerous studies had established the theory of HLA-1 restricted and cytotoxic T lymphocytes mediated hepatocytolysis. Recent immunological study also found the relationship between HBeAg seroconversion and IL-10/ IL-12[30] and IL-21[31]; chemokine (C-X-C motif) ligand CXCL-9 and CXCL-10 also play a major role in the development of CHB with acute exacerbation [32]. Those immunological responses had been identified by hepatic histological studies, serological studies and blood cellular studies. However, lymph nodal reaction, a symbol of immune response, in relation to HBeAg seroconversion, has not been studied before. Moreover, the celiac nodes were proven by studies to be an important lymphatic drainage path of the liver tissues by using purified dendritic cells and orally administered antigens able to trigger antigen-specific regulatory T-cells in the celiac lymph node [33–35]. Then, Zheng et al provided evidence via hydrodynamic HBV plasmid injection that liver-draining lymph nodes induce an anti-HBV specific immune response responsible for HBV clearance [36]. Our study found that perihepatic lymph nodes in CHB with acute exacerbation will increase in sizes(70%), indicating a robust immune reaction in the liver, which in turn support the importance of immune response in CHB with acute exacerbation.

Little research is found on relationship of hepatitis B status and lymph node size. In 2001 Choi first reported 96%[37] CHB had a lymph node enlargement phenomenon related to the degree of liver inflammation, but not to the degree of liver fibrosis and hepatitis B viral load. In 2006, we noticed that about 60% [24] HBsAg carriers have enlarged lymph nodes which was linearly related to ALT values. Recently in 2013, Shu found that enlarged lymph node could be found in about 90% of CHB patients by using MRI and also used at least two lymph nodes with short diameter> 5mm and nodal size index (product of the long and short axes) > 180 mm^2^ to predict the degree of liver inflammation (≧grading 2) [38], which further support that nodal size and numbers are related to the degree of liver inflammation (grading). Our previous studies comparing various methods found that short diameter> 5mm is the simplest method for measuring lymph node size and also has its clinical significance[24,39]. In this study, we found that short diameter and volume can both represent reactive lymph nodes (Table 1). Therefore, we believed that lymph nodes around the common hepatic artery that have a short diameter> 5mm represent liver inflammation in hepatitis C or hepatitis B patients; and an enlarged perihepatic lymph node depicted during routine abdominal ultrasonography in a healthy or acutely ill individual should prone one to survey possible acute hepatitis flare.

In our study, the incidence of enlarged lymph nodes (width > = 5mm) and the nodal size were higher in HBeAg positive group than HBeAg negative group. The immunopathogenic mechanisms of HBeAg negative CHB have not been elucidated with studies supporting same immune responses in HBeAg positive and negative patients[40] and those favoring different serum cytokine expressions in the two subsets[41]. Our sonographic finding might also indicate a different immune response in the two important clinical subsets, which warrant further study. In addition, we also found that in HBeAg positive patients, lymph node enlargement is more common in spontaneous flare group than in antiviral withdrawal group (p<0.05) (Table 6). This result may partly explain the higher durability of HBeAg seroconversion after spontaneous flare when compared to that after antiviral withdrawal flare[42].

It would be clinically useful to predict HBeAg seroconversion, because antiviral treatments can be withheld in the patients in whom HBeAg disappears and anti-HBe develops spontaneously. As mentioned before, many indicators can predict HBeAg seroconversion. However, most studies rely on performance of viral protein in liver cells, antigen reaction in peripheral blood mononuclear cells (PBMC) and changes in serum cytokines/chemokines and single nucleotide polymorphism genotyping. Most of these indicators are processed in the laboratory and more time consuming than a quick clinical sonography exam. Through the observation of nodal size during acute flare stage, we can speculate the strength of immune response and predict the occurrence of HBeAg seroconversion, and save the expense of antiviral therapy. Besides, interval nodal size change between acute flare phase and recovery phase further enhance the predictive effect. If setting the acute flare stage lymph node width≧8mm as standard alone, there was 72% of positive prediction rate of HBeAg seroconversion; if setting interval nodal change ≧3mm as standard, there was 68% of positive prediction rate of HBeAg seroconversion; if setting both as standard, there was 75% of positive prediction rate of HBeAg seroconversion. If neither standards were met, only 22% will proceed to HBeAg seroconversion (p<0.001) (Table 6). Changes in nodal size during acute flare phase and recovery phase may provide a reference for clinicians in decision making of oral antiviral prescription.

There are some limitations in the present study. First, in this retrospective analysis, data of HBV genotype, a well-established predictor of HBeAg seroconversion are inadequate. Only 34 (39%) subjects have data of HBV genotype (B:C = 18:16). Most of the group 2 patients are genotype B and all subjects of group 3 are genotype C. Second, there are no data to correlate the nodal sizes and serum immune profiles, which will be our next objective.

## Conclusion

We herein first present the ultrasonographic sign of chronic hepatitis B with acute exacerbation by detection of perihepatic lymph nodes. The nodal sizes are more prominent on HBeAg- positive subjects than those of HBeAg- negative subjects. Those with HBeAg seroconversion had larger lymph node then those without HBeAg seroconversion. And the nodal size change between acute flare phase and recovery phase also helps predict HBeAg seroconversion, which is a critical point in both the natural course and in antiviral therapy. The nodal reactions correlate with the host immune response on chronic B hepatitis and are worth further exploration.
